# Supplementary material for: MRI Liver Imaging Integrated with Texture Analysis in Native Liver Survivor Patients with Biliary Atresia after Kasai Portoenterostomy: Correlation with Medical Outcome after Surgical Treatment
Source: Bioengineering (Basel). 2023 Feb 28;10(3):306. doi: 10.3390/bioengineering10030306 (PMC10045135; doi:10.3390/bioengineering10030306)
Supplement: Supplementary file 1 [file bioengineering-10-00306-s001.zip › bioengineering-2175938-supplementary.pdf]

Supplementary Table 1. List of the 25 gray-level co-occurrence matrix texture features calculated from the Apparent Diffusion Coefficient map images, with mean values and standard deviations reported for the two patients' groups.

**Feature name**	**Mean ± SD (Group 1)**	**Mean ± SD (Group 2)**	**P value**
Joint maximum_Co-occurrence matrix (2D, averaged)	0.11±0.02	0.12±0.03	0.57
Joint average_Co-occurrence matrix (2D, averaged)	4.27±0.30	4.26±0.45	0.98
Joint variance_Co-occurrence matrix (2D, averaged)	3.29±0.85	3.27±0.49	0.95
Joint entropy_Co-occurrence matrix (2D, averaged)	4.64±0.23	4.62±0.20	0.79
Difference average_Co-occurrence matrix (2D, averaged)	0.90±0.10	0.88±0.12	0.67
Difference variance_Co-occurrence matrix (2D, averaged)	0.61±0.09	0.61±0.11	0.90
Difference entropy_Co-occurrence matrix (2D, averaged)	1.58±0.10	1.57±0.11	0.79
Sum average_Co-occurrence matrix (2D, averaged)	8.53±0.60	8.53±0.91	0.98
Sum variance_Co-occurrence matrix (2D, averaged)	11.67±3.23	11.63±1.71	0.97
Sum entropy_Co-occurrence matrix (2D, averaged)	3.56±0.14	3.56±0.10	0.91
Angular second moment_Co-occurrence matrix (2D, averaged)	0.05±0.01	0.05±0.01	0.73
Contrast_Co-occurrence matrix (2D, averaged)	1.48±0.25	1.45±0.33	0.76
Dissimilarity_Co-occurrence matrix (2D, averaged)	0.90±0.10	0.88±0.12	0.67
Inverse difference_Co-occurrence matrix (2D, averaged)	0.64±0.03	0.64±0.03	0.63
Inverse difference normalised_Co-occurrence matrix (2D, averaged)	0.91±0.01	0.91±0.01	0.65
Inverse difference moment_Co-occurrence matrix (2D, averaged)	0.61±0.04	0.61±0.04	0.62
Inverse difference moment normalised_Co-occurrence matrix (2D, averaged)	0.98±0.01	0.98±0.01	0.74
Inverse variance_Co-occurrence matrix (2D, averaged)	0.50±0.02	0.50±0.02	0.93
Correlation_Co-occurrence matrix (2D, averaged)	0.76±0.04	0.77±0.03	0.54
Autocorrelation_Co-occurrence matrix (2D, averaged)	20.91±2.61	21.09±3.85	0.88
Cluster tendency_Co-occurrence matrix (2D, averaged)	11.67±3.23	11.63±1.71	0.97
Cluster shade_Co-occurrence matrix (2D, averaged)	5.31±9.33	5.81±11.65	0.90
Cluster prominence_Co-occurrence matrix (2D, averaged)	320.64±130.43	330.56±68.61	0.82
Information correlation 1_Co-occurrence matrix (2D, averaged)	-0.31±0.03	-0.31±0.03	0.56
Information correlation 2_Co-occurrence matrix (2D, averaged)	0.89±0.02	0.89±0.02	0.77

Supplementary Table 2. List of the 25 gray-level co-occurrence matrix texture features calculated from the T2-weighted images, with mean values and standard deviations reported for the two patients' groups.

**Feature name**	**Mean ± SD (Group 1)**	**Mean ± SD (Group 2)**	**P value**
Joint maximum_Co-occurrence matrix (2D, averaged)	0.13±0.04	0.13±0.04	0.97
Joint average_Co-occurrence matrix (2D, averaged)	4.01±0.41	4.01±0.37	0.98
Joint variance_Co-occurrence matrix (2D, averaged)	2.97±0.39	2.92±0.41	0.77
Joint entropy_Co-occurrence matrix (2D, averaged)	4.60±0.26	4.63±0.18	0.71
Difference average_Co-occurrence matrix (2D, averaged)	0.93±0.19	1.01±0.16	0.26
Difference variance_Co-occurrence matrix (2D, averaged)	0.80±0.23	0.82±0.18	0.74
Difference entropy_Co-occurrence matrix (2D, averaged)	1.67±0.19	1.73±0.15	0.40
Sum average_Co-occurrence matrix (2D, averaged)	8.02±0.83	8.01±0.73	0.98
Sum variance_Co-occurrence matrix (2D, averaged)	10.13±1.34	9.77±1.89	0.53
Sum entropy_Co-occurrence matrix (2D, averaged)	3.47±0.09	3.43±0.09	0.28
Angular second moment_Co-occurrence matrix (2D, averaged)	0.06±0.02	0.06±0.01	0.94
Contrast_Co-occurrence matrix (2D, averaged)	1.75±0.59	1.93±0.51	0.39
Dissimilarity_Co-occurrence matrix (2D, averaged)	0.93±0.19	1.01±0.16	0.26
Inverse difference_Co-occurrence matrix (2D, averaged)	0.64±0.05	0.62±0.04	0.21
Inverse difference normalised_Co-occurrence matrix (2D, averaged)	0.90±0.02	0.90±0.01	0.23
Inverse difference moment_Co-occurrence matrix (2D, averaged)	0.61±0.06	0.58±0.05	0.20
Inverse difference moment normalised_Co-occurrence matrix (2D, averaged)	0.98±0.01	0.97±0.01	0.34
Inverse variance_Co-occurrence matrix (2D, averaged)	0.47±0.03	0.47±0.03	0.89
Correlation_Co-occurrence matrix (2D, averaged)	0.70±0.09	0.65±0.08	0.16
Autocorrelation_Co-occurrence matrix (2D, averaged)	18.45±3.32	18.25±2.80	0.86
Cluster tendency_Co-occurrence matrix (2D, averaged)	10.13±1.34	9.77±1.59	0.53
Cluster shade_Co-occurrence matrix (2D, averaged)	9.90±11.30	10.22±10.57	0.94
Cluster prominence_Co-occurrence matrix (2D, averaged)	269.30±62.01	260.55±90.01	0.78
Information correlation 1_Co-occurrence matrix (2D, averaged)	-0.27±0.05	-0.26±0.05	0.39
Information correlation 2_Co-occurrence matrix (2D, averaged)	0.86±0.03	0.84±0.04	0.24
